# The copper chelator ATN-224 induces caspase-independent cell death in diffuse large B cell lymphoma

**DOI:** 10.3892/ijo.2014.2396

**Published:** 2014-04-23

**Authors:** KRISTY LEE, MATTHEW R. HART, MARGARET M. BRIEHL, ANDREW P. MAZAR, MARGARET E. TOME

**Affiliations:** 1 Departments of Pathology, University of Arizona, Tucson, AZ 85724; 2 GIPD Genetics, University of Arizona, Tucson, AZ 85724; 3 Medical Pharmacology, University of Arizona, Tucson, AZ 85724; 4 Department of Life Processes Institute, Northwestern University, Evanston, IL 85724, USA

**Keywords:** Bcl-2, Bcl-xL, Mcl-1, lymphoma, cytochrome *c* oxidase, mitochondria

## Abstract

Bcl-2 and other anti-apoptotic proteins are associated with defective caspase-dependent apoptotic pathways, resulting in chemoresistance. We have previously shown that ATN-224, a copper chelator drug, induces cell death in murine thymic lymphoma cells transfected with Bcl-2. In the current study, we tested whether ATN-224 was effective in diffuse large B cell lymphoma (DLBCL) cells, which have increased anti-apoptotic proteins through translocation or amplification. We found that nanomolar concentrations of ATN-224 induced cell death in DLBCL cells independent of Bcl-2, Bcl-xL or Mcl-1 status. ATN-224 treatment resulted in mitochondrial dysfunction, release of apoptosis-inducing factor (AIF) and induction of caspase-independent cell death. In addition, ATN-224 degraded Mcl-1 and enhanced the effect of the BH3 mimetic ABT-263. These findings indicate that ATN-224 has potential as a therapeutic for the treatment of DLBCL. Induction of caspase-independent cell death in apoptosis-resistant DLBCL would provide a therapeutic alternative for the treatment of refractory disease.

## Introduction

Diffuse large B cell lymphoma (DLBCL) is the most common diagnosed form of non-Hodgkin lymphoma (NHL). While cure rates have increased, the lack of response to current standard treatment and/or relapse leaves approximately 50% of patients incurable (1,2). Many of the cytotoxic drugs used to treat DLBCL induce caspase-dependent apoptosis, however, a significant number of patients acquire resistance that is associated with defective caspase-dependent apoptotic pathways (3). Upregulation of the *BCL2* gene, due to either the t(14;18) translocation or genomic *BCL2* gain/amplification, results in overexpression of the Bcl-2 protein and apoptosis resistance (2). The Bcl-2 family is a group of mitochondrial-associated proteins that are characterized as either anti-apoptotic or pro-apoptotic. The canonical function of Bcl-2 and other anti-apoptotic proteins, such as Bcl-xL and Mcl-1, is to prevent mitochondrial outer membrane permeabilization (MOMP) (4). Pro-apoptotic proteins, such as BH3-only proteins, bind to and inhibit the function of anti-apoptotic proteins to induce MOMP (5). There are several BH3 mimetic drugs that are currently being developed and are in clinical trials (6). While these drugs are promising, resistance through the upregulation of Mcl-1 is a problem (7). Due to their different binding affinities, a combination of BH3 mimetics that target different anti-apoptotic proteins would be necessary to achieve an optimal therapeutic effect (6).

The non-canonical function of Bcl-2 and other anti-apoptotic proteins is to maintain mitochondrial homeostasis by regulating mitochondrial membrane potential (*ΔΨ_m_*) and/or mitochondrial respiration (8–10). Regulation of these mitochondrial processes may or may not contribute to the ability of anti-apoptotic proteins to prevent apoptosis. In response to cellular stress, Chen and Pervaiz showed that Bcl-2 alters the activity of the copper-dependent enzyme cytochrome *c* oxidase (CcOX), the terminal subunit of the mitochondrial respiratory chain (11). Data from their study suggest that the ability to modulate CcOX activity contributes to the ability of Bcl-2 to inhibit caspase-dependent cell death. We recently showed that ATN-224 (choline tetrathiomolybdate), a copper chelator drug, inhibits CcOX, causing mitochondrial dysfunction. Treatment with ATN-224 resulted in cell death in parental murine thymic lymphoma cells and those transfected with Bcl-2 that are apoptosis resistant (12). The ability of ATN-224 to induce cell death in an isogenic cell model overexpressing Bcl-2 led to the hypothesis that ATN-224 treatment would be effective in DLBCL cells with upregulated Bcl-2.

In this study, we tested whether using ATN-224 to target the non-canonical function of Bcl-2 and other anti-apoptotic proteins could induce cell death in DLBCL cells independent of the level of anti-apoptotic proteins. We show that nanomolar concentrations of ATN-224 can induce caspase-independent cell death via release of apoptosis inducing factor (AIF). ATN-224 also enhanced the overall effect of the BH3 mimetic, ABT-263, in apoptosis-resistant DLBCL. Taken together these data suggest that ATN-224 has therapeutic potential as a single agent or as an adjuvant, specifically in patients with apoptosis-resistant disease.

## Materials and methods

### Drug treatments and reagents

ATN-224 was provided by Dr Andrew Mazar (Northwestern University, Evanston, IL). ABT-263 and ABT-737 were purchased from ChemieTek (Indianapolis, IN). ZVAD-FMK was purchased from Enzo Life Sciences (Plymouth Meeting, PA). All other drugs and chemicals were purchased from Sigma Chemical Co. (St. Louis, MO) unless otherwise stated.

### Cell lines

SUDHL-4, SUDHL-10, U-2932 cells and Granta 519 cells were obtained from the Arizona Lymphoid Tissue and Blood Repository (University of Arizona, Tucson, AZ). SUDHL8 and SUDHL4 R2 cells were obtained from Dr Anthony Letai (Dana-Farber Cancer Institute, Boston, MA). All cells were maintained in suspension in RPMI-1640 (Cellgro; Mediatech, Manassas, VA) supplemented with 10% fetal bovine serum (Gemini, Sacramento, CA), 2 mM L-glutamine and 50 U/ml each of penicillin and streptomycin (all from Invitrogen, Carlsbad, CA) at 37°C in a 5% CO_2_ humidified environment. SUDHL-4 R2 cell cultures were supplemented with 5 *μ*g/ml verapamil and 1 *μ*M ABT-737, as previously described (7).

### Cell viability measurements

The number of viable treated cells, relative to control treated cells, was measured after 72 h of treatment using the Non-radioactive Cell Proliferation Assay (MTS) according to the manufacturer’s instructions (Promega Corp., Madison, WI). Absorbance was read at 490 nm using a Synergy HT plate reader (BioTek Instruments, Winooski, VT). The MTS assay was used to determine the estimated ATN-224 concentration needed to decrease the number of viable cells by 50 (EC_50_) and 25% (EC_25_). Viable cell number was also determined by propidium iodide (PI) (Molecular Probes, Eugene, OR) uptake, as previously described (12). PI uptake was used to determine the effect of drugs alone or in combination with ATN-224. Caspase 3 activity was measured using Ac-DEVD-p-nitroanilide (pNA) (Enzo Life Sciences), as previously described (13).

### ΔΨ_m_

The fluorescent probe JC-1 (Molecular Probes) was used to measure *ΔΨ_m_*. Cells were incubated with 2 *μ*g/ml JC-1 for 30 min at 37°C in a 5% CO_2_ humidified environment. Cells were then washed with PBS, resuspended in PBS and transferred to a black well plate. JC-1 J-aggregates (Ex: 560/Em: 595) were measured using a Synergy HT plate reader (Bio Tek Instruments). Fluorescence was normalized to cellular protein.

### Nuclear condensation

Following treatment, cells were transferred to poly-L-lysine coated chamber slides. Chambers were removed from the slide, coated with mounting medium containing Dapi (Vectashield; Vector Laboratories, Burlingame, CA) and cover slips applied. Slides were visualized using the Olympus Fluorview FV1000 Confocal Microscope with FV10-ASW software (Olympus America Inc., San Jose, CA).

### Immunoblot analysis

Protein fractions isolated with the Mitochondrial Isolation kit for Cultured Cells (Thermo Fisher Scientific, Waltham, MA) or total cell lysates were separated by SDS-PAGE and transferred to a PVDF membrane using standard protocols. Blots were probed with antibodies for Mcl-1, AIF, COX IV (Cell Signaling, Danvers, MA), cyto-chrome *c*, Bcl-2, Bcl-xL, Bak, Bax, Bim (BD Pharmingen, San Diego, CA), Bid (Abcam, Cambridge, MA) or Noxa (Imgenex, San Diego, CA). Proteins were detected with either horseradish peroxidase-linked anti-rabbit Ig or horse-radish peroxidase-linked anti-mouse Ig (Cell Signaling), where appropriate, and visualized by chemiluminescence (Perkin-Elmer, Waltham, MA). Blots were also probed with anti-β-actin (Abcam) as a loading control. Restore Western Blot Stripping Buffer (Thermo Scientific) was used to visualize multiple bands on the same blot. Film was scanned, and images were cropped to show the bands of interest then contrast adjustments were made to the cropped image. Bands were quantified using Image J (NIH).

### Statistics

Means were compared using Student’s t-test with the algorithm in Excel (Microsoft Corp., Redmond, WA). Means were considered significantly different when p≤0.05. When a comparison required multiple t-tests, the Dunn-Bonferroni method was used to control for type I error (14).

## Results

### Apoptosis-resistant cells are sensitive to ATN-224

**Figure 1.**
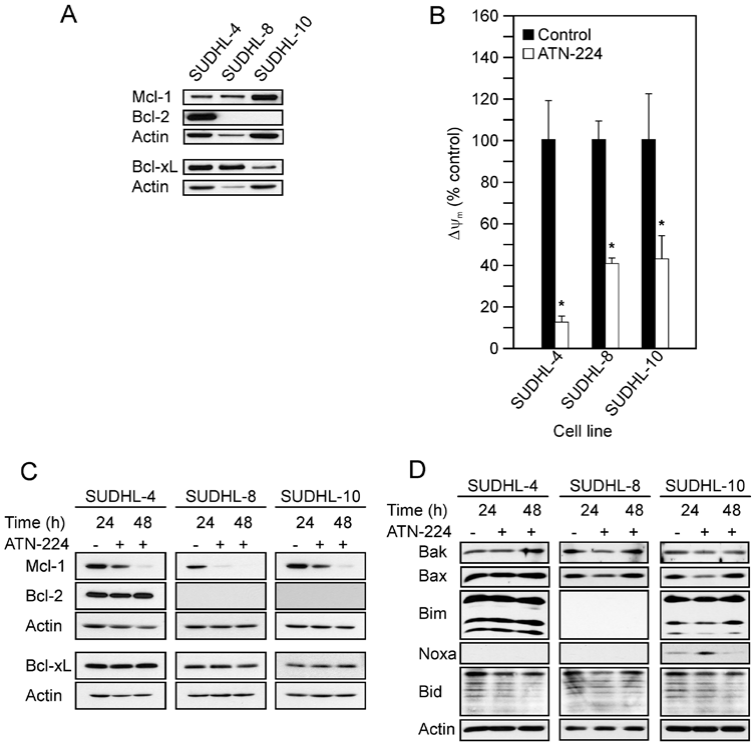
ATN-224 effects the mitochondria. (A) Immunoblots showing Mcl-1, Bcl-2 and Bcl-xL protein levels in SUDHL-4, SUDHL-8 and SUDHL-10 cells. Immunoblots showing actin protein levels to demonstrate similar loading. (B) JC-1 aggregates (*ΔΨ_m_*) in SUDHL-4, SUDHL-8 and SUDHL-10 cells treated with vehicle or ATN-224 (EC_50_) for 12 h. All values are mean ± SEM (n ≥3). ^*^p≤0.05, significantly different from vehicle treated control cells. (C) Immunoblots showing Mcl-1, Bcl-2 and Bcl-xL protein levels in SUDHL-4, SUDHL-8 and SUDHL-10 cells treated with vehicle or ATN-224 (EC_50_) for 24 and 48 h. Immunoblots showing actin protein levels to demonstrate similar loading. (D) Immunoblots showing relative Bak, Bax, Bim, Noxa and Bid protein levels. Immunoblots showing actin as a loading control. All immunoblots are representative blots from three independent sample collections.

To determine the ability of ATN-224 to overcome apoptosis-resistance, we characterized three DLBCL cell lines for the anti-apoptotic proteins, Bcl-2, Bcl-xL and Mcl-1. The immunoblots in Fig. 1A show the following: the SUDHL-4 had high levels of Bcl-2 and Bcl-xL, with moderate levels of Mcl-1; the SUDHL-8 had high levels of Bcl-xL and moderate levels of Mcl-1; the SUDHL-10 had high levels of Mcl-1 and moderate levels of Bcl-xL. Taken together all three DLBCL cell lines displayed various levels of anti-apoptotic proteins, which contribute to apoptosis-resistance.

To establish whether the DLBCL cells were sensitive to ATN-224, we measured cell viability following ATN-224 treatment. In the SUDHL-4, SUDHL-8 and SUDHL-10 cells, nanomolar concentrations of ATN-224 decreased the number of viable cells (Table I). Recent studies suggest that the protective function of Bcl-2, in part, is due to its ability to regulate mitochondrial respiration (9). In previous studies we have shown that ATN-224 inhibits CcOX and decreases *ΔΨ_m_* in murine thymic lymphoma cells that overexpress Bcl-2 (12). To determine whether ATN-224 is targeting the mitochondria, we assessed *ΔΨ_m_* following ATN-224 treatment. In the SUDHL-4, SUDHL-8 and SUDHL-10 cells, ATN-224 treatment significantly decreased *ΔΨ_m_* at 12 h (Fig. 1B).

In previous studies, we have shown that ATN-224 inhibits superoxide dismutase 1 (SOD1), a copper-dependent enzyme responsible for the detoxification of superoxide, resulting in increased levels of superoxide (12). An increase in oxidants causes Bcl-2 degradation through the ubiquitin-proteasomal pathway in other cell types (15). In response to ATN-224 treatment, Bcl-2, Bcl-xL and Mcl-1 are unable to maintain mitochondrial homeostasis, as indicated by the decrease in *ΔΨ_m_*, suggesting that they may be indirect targets of ATN-224. To characterize the effect of ATN-224 on Bcl-2 and other anti-apoptotic proteins, we measured Bcl-2, Bcl-xL and Mcl-1 protein levels following ATN-224 treatment. In the SUDHL-4, SUDHL-8 and SUDHL-10 cells, we detected decreases in Mcl-1, but no change in Bcl-2 or Bcl-xL protein levels (Fig. 1C). Taken together these results indicate that ATN-224 treatment induces mitochondrial dysfunction, independent of Bcl-2, Bcl-xL and Mcl-1 status, which may contribute to the ATN-224 sensitivity of apoptosis-resistant cells.

The decision to undergo apoptosis is influenced by the balance between the anti- and pro-apoptotic Bcl-2 family members. To determine whether, in addition to the loss of Mcl-1, ATN-224 treatment increased pro-apoptotic Bcl-2 family member proteins we measured the levels of the pro-apoptotic proteins Bak, Bax, Bim, Noxa and Bid. As shown in Fig. 1D, we did not see a consistent increase in any of the pro-apoptotic proteins we measured across the cell types and in several cases there was a decrease, e.g., Bax in the SUDHL-8 and SUDHL-10 cells. We did see an increase in Noxa after a 24 h-ATN-224 treatment in the SUDHL-10; Noxa was undetectable in the other two cell types. These data suggest that the primary effect of ATN-224 on Bcl-2 family member protein levels is a decrease in Mcl-1. A decrease in Mcl-1 could tip the balance toward apoptosis.

### ATN-224 induces caspase-independent cell death in apoptosis-resistant cells

**Figure 2.**
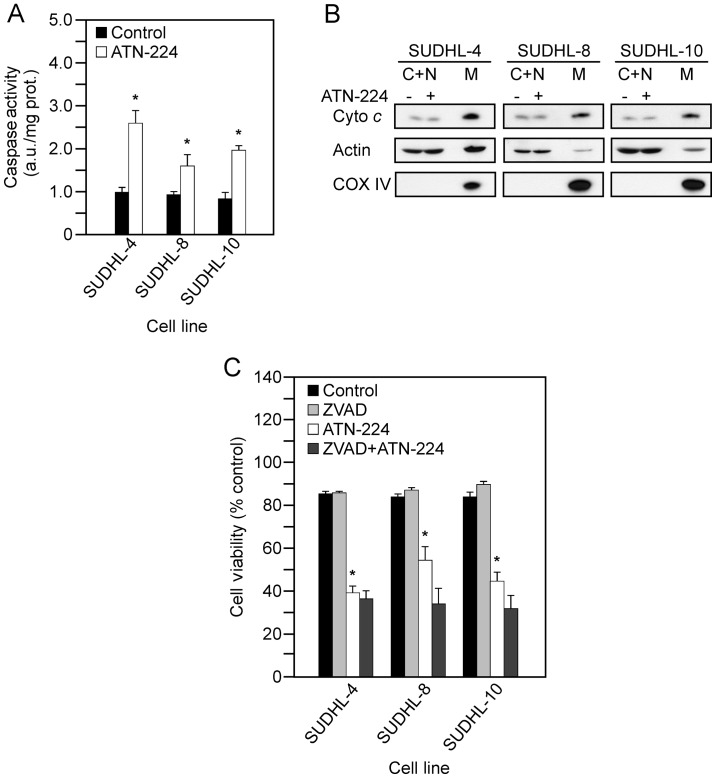
ATN-224 induces caspase-independent cell death. (A) Caspase 3 activity measured in SUDHL-4, SUDHL-8 and SUDHL-10 cells treated with vehicle or ATN-224 (EC_50_) for 24 h. (B) Immunoblots showing cytochrome c release from the mitochondria in SUDHL-4, SUDHL-8 and SUDHL-10 cells treated with vehicle or ATN-224 (EC_50_) for 24 h. C+N, cytosolic and nuclear fraction; M, mitochondrial loading control. Immunoblots showing actin demostrate similar loading. Immunoblots showing COX IV demostrate the integrity of the mitochondrial prep. All immunoblots are representative blots from three independent sample collections. (C) Cell viability in SUDHL-4, SUDHL-8 and SUDHL-10 cells treated with vehicle, 2 *μ*M ZVAD-FMK (ZVAD), ATN-224 (EC_50_) or a combination of ZVAD-FMK and ATN-224 for 72 h. All values are mean ± SEM (n ≥3). ^*^p≤0.05, significantly different from vehicle treated control cells.

The intrinsic apoptotic pathway involves the formation of MOMP and the release of cytochrome *c* from the mitochondria, which leads to caspase 3 activation and cell death (5). The upregulation of anti-apoptotic proteins, such as Bcl-2, Bcl-xL and Mcl-1, prevent the formation of MOMP (4). To determine whether ATN-224 treatment results in apoptosis, we measured cytochrome *c* release from the mitochondria and caspase 3 activity following ATN-224 treatment. In the SUDHL-4, SUDHL-8, SUDHL-10 cells, we measured significant increases in caspase 3 activity (Fig. 2A), but detected no significant increase in cytochrome *c* release from the mitochondria (Fig. 2B). To determine whether ATN-224 induced cell death is caspase-dependent, we used the pan caspase inhibitor, ZVAD-FMK, in combination with ATN-224 and measured cell viability. In the SUDHL-4, SUDHL-8 and SUDHL-10 cells, the addition of ZVAD-FMK was unable to attenuate the effect of ATN-224 (Fig. 2C). These results indicate that ATN-224 induced cell death is caspase-independent.

### ATN-224 induces AIF release and nuclear condensation in apoptosis-resistant cells

**Figure 3.**
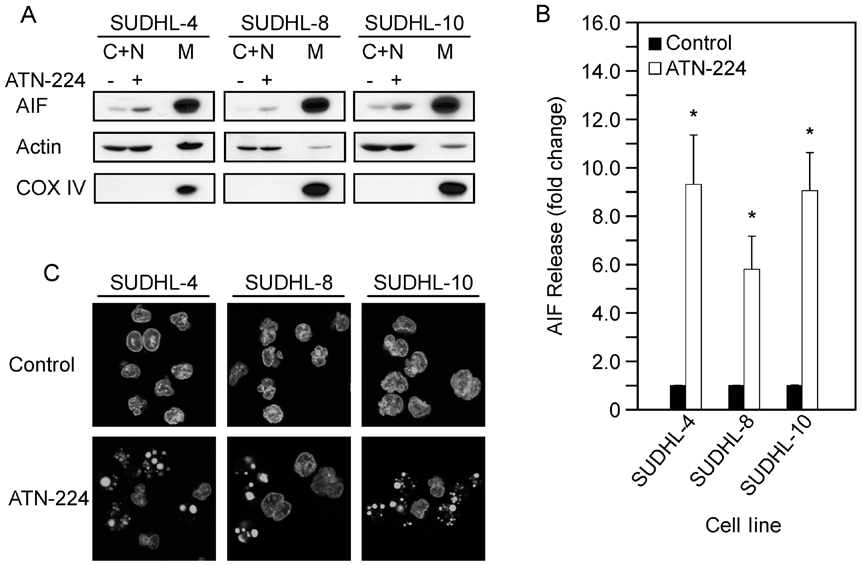
ATN-224 induces AIF release from the mitochondria. (A) Immunoblots showing AIF release from the mitochondria in SUDHL-4, SUDHL-8 and SUDHL-10 cells treated with vehicle or ATN-224 (EC_50_) for 24 h. C+N, cytosolic and nuclear fraction; M, mitochondrial loading control. Immunoblots showing actin demostrate similar loading. Immunoblots showing COX IV demostrate the integrity of the mitochondrial prep. All immunoblots are representative blots from three independent sample collections. (B) Quantification of AIF release from the mitochondria. (C) Nuclear condensation in SUDHL-4, SUDHL-8 and SUDHL-10 cells treated with vehicle or ATN-224 (EC_50_) for 24 h. All values are mean ± SEM (n ≥3). ^*^p≤0.05, significantly different from ATN-224 treated control cells.

We have shown that ATN-224 targets the mitochondria and induces caspase-independent cell death. The mitochondria contain other death inducing proteins, such as apoptosis inducing factor (AIF). The release of AIF from the mitochondria results in the activation of caspases and nuclear condensation, however, AIF-induced cell death is caspase-independent (16). To determine whether AIF is involved in ATN-224-induced cell death, we measured AIF release from the mitochondria following ATN-224 treatment. In the SUDHL-4, SUDHL-8 and SUDHL-10 cells, we detected a significant increase in AIF release from the mitochondria (Fig. 3A and B). To further assess the involvement of AIF, we looked for nuclear condensation. In the SUDHL-4, SUDHL-8 and SUDHL-10 cells, we detected nuclear condensation following ATN-224 treatment (Fig. 3C). These results indicate that ATN-224 induces AIF release and nuclear condensation. These data suggest that ATN-224 induces cell death via a mechanism that circumvents apoptosis resistance.

### ATN-224 induces cell death in aggressive lymphomas

**Figure 4.**
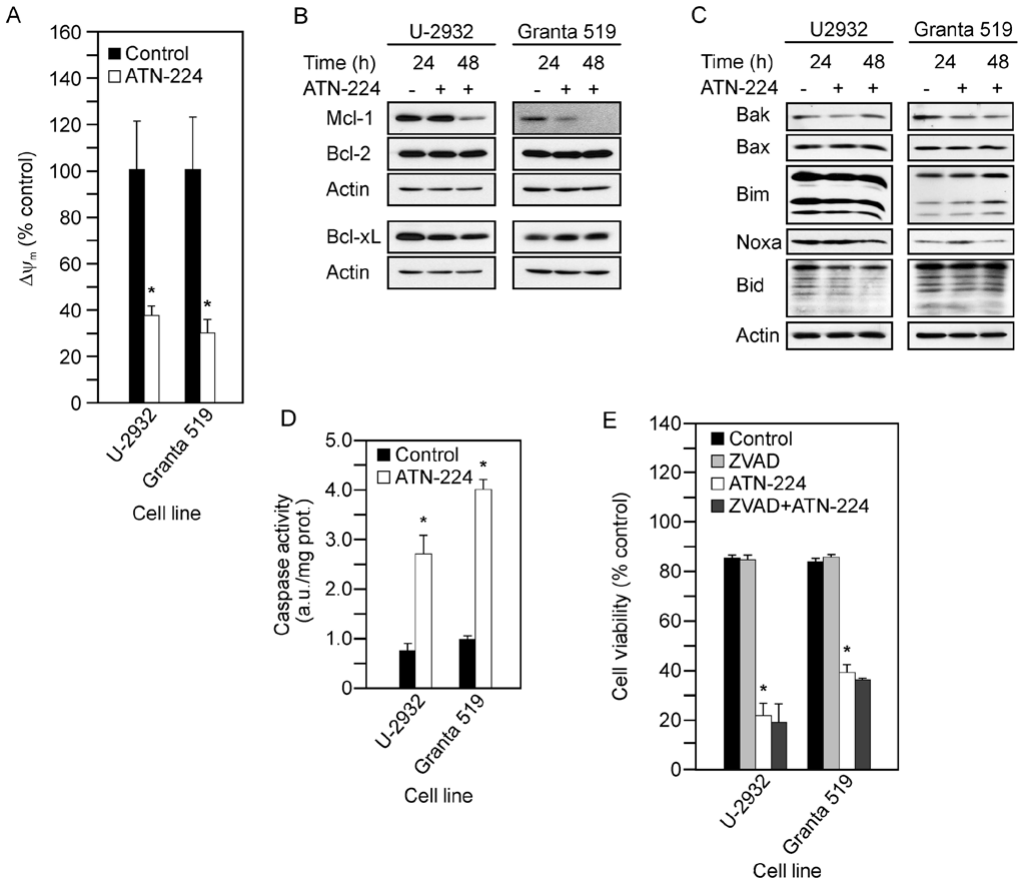
ATN-224 in aggressive lymphomas. (A) JC-1 aggregates (*ΔΨ_m_*) in U-2932 and Granta 519 cells treated with vehicle or ATN-224 (EC_50_) for 12 h. (B) Immunoblots showing Mcl-1, Bcl-2 and Bcl-xL protein levels in U-2932 and Granta 519 cells treated with vehicle or ATN-224 (EC_50_) for 24 and 48 h. Immunoblots showing actin protein levels to demonstrate similar loading. (C) Immunoblots showing Bak, Bax, Bim, Noxa and Bid protein levels in U-2932 and Grants 519 cells with the same treatments as in (B). All immunoblots are representative blots from three independent sample collections. (D) Caspase 3 activity measured in U-2932 and Granta 519 cells treated with vehicle or ATN-224 (EC_50_) for 24 h. (E) Cell viability (PI uptake) in U-2932 and Granta 519 cells treated with vehicle, 25 *μ*M ZVAD-FMK (ZVAD), ATN-224 (EC_50_) or a combination of ZVAD-FMK and ATN-224 for 72 h. All values are mean ± SEM (n ≥3). ^*^p≤0.05, significantly different from vehicle treated control cells.

The t(14;18) translocation or genomic gain/amplification causes *BCL2* upregulation. *BCL2* translocations are commonly associated with follicular lymphoma and the DLBCL germinal center B-cell-like (GBC) subtype (17). *BCL2* gain/amplification are commonly associated with the DLBCL activated B-cell-like (ABC) subtype and mantle cell lymphoma (MCL), two aggressive types of NHL (18,19). We have shown that ATN-224 treatment circumvents Bcl-2 overexpression and induces death in the SUDHL-4 cells, which have the t(14;18) translocation (20). To determine whether our findings extend to aggressive NHLs that have *BCL2* gain/amplification, we used the U-2932 and Granta 519, a DLBCL and an MCL cell line, respectively, which have *BCL2* gain/amplification (21,22). We first measured cell viability to establish whether the U-2932 and Granta 519 cells were sensitive to ATN-224 (Table I). To determine whether ATN-224 treatment had an effect in the U-2932 and Granta 519 cells similar to the effect in the SUDHL-4, SUDHL-8 and SUDHL-10 cells, we measured the following: *ΔΨ_m_*; Mcl-1, Bcl-2 and Bcl-xL protein levels; pro-apoptotic Bcl-2 family member protein levels; and caspase 3 activity. Following ATN-224 treatment in the U-2932 and Granta 519 cells, we detected: i) decreases in *ΔΨ_m_* at 12 h (Fig. 4A); ii) decreases in Mcl-1 and no change in Bcl-2 or Bcl-xL protein levels (Fig. 4B); iii) no consistent change in pro-apoptotic Bcl-2 family member protein levels, although similar to the SUDHL-10 cells, Granta 519 cells showed a slight increase in Noxa after 24 h (Fig. 4C); and significant increases in caspase 3 activity (Fig. 4D). To confirm that the ATN-224-induced cell death was caspase-independent, we measured cell viability using the pan-caspase inhibitor, ZVAD-FMK, in combination with ATN-224. In both the U-2932 and Granta 519 cells, the addition of ZVAD-FMK was unable to attenuate the effect of ATN-224 (Fig. 4E). Taken together, these data suggest ATN-224 circumvents the overexpression of Bcl-2, regardless of the mechanism by which *BCL2* is upregulated, and has therapeutic potential in the treatment of aggressive NHL.

### ATN-224 induces cell death in ABT-737-resistant cells and enhances the effect of ABT-263

**Figure 5.**
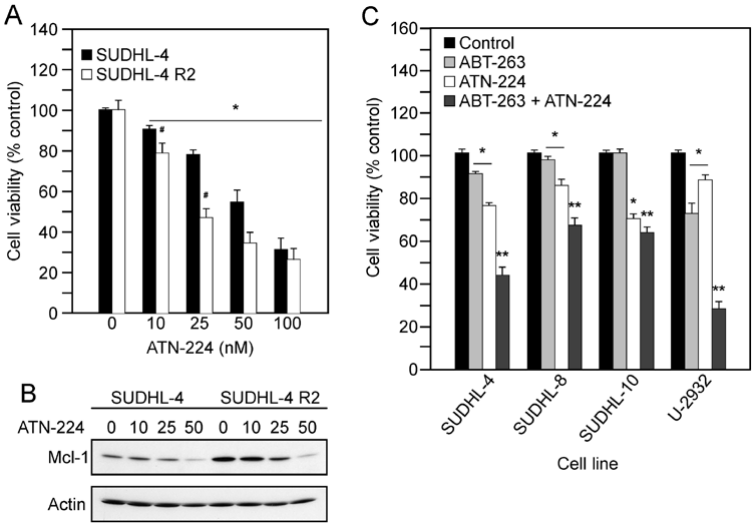
ATN-224 enhances the effect of BH3 mimetics. (A) Cell viability in SUDHL-4 and SUDHL-4 R2 cells treated with vehicle or ATN-224 (10, 25, 50 and 100 nM) for 72 h. (B) Immunoblot showing Mcl-1 protein levels in SUDHL-4 and SUDHL-4 R2 cells treated with vehicle or ATN-224 (10, 25 and 50 nM) for 24 h. Immunoblot showing actin protein levels to demonstrate similar loading. All immunoblots are representative blots from three independent sample collections. (C) Cell viability in SUDHL-4, SUDHL-8, SUDHL-10 and U-2932 cells treated with vehicle, 250 nM ABT-263, ATN-224 (EC_25_) or a combination of ABT-263 and ATN-224 for 72 h. All values are mean ± SEM (n ≥3). ^*^p≤0.05, significantly different from vehicle treated control cells. ^**^p≤0.01, significantly different from ABT-263 or ATN-224 treated cells. ^#^p≤0.05, significantly different from SUDHL-4 ATN-224 treated cells.

Drugs targeting Bcl-2 and Bcl-xL, such as ABT-737 and ABT-263 derivatives, are currently in clinical trials. Yecies *et al* showed that SUDHL-4 R2 cells, selected for resistance to ABT-737, upregulate Mcl-1 (7). The ability of ATN-224 to degrade Mcl-1 suggests that the SUDHL-4 R2 cells may be sensitive to ATN-224. To determine the effect of ATN-224 on SUDHL-4 R2 cells, we measured cell viability following treatment with various concentrations of ATN-224. In the SUDHL-4 and SUDHL4-R2 cells, we measured a significant decrease in the number of viable cells, which appears to be concentration-dependent (Fig. 5A). We also detected decreases in Mcl-1 protein levels (Fig. 5B). These data suggest ATN-224 has the potential to overcome Mcl-1 resistance, thus sensitizing cells to drugs that target Bcl-2 and Bcl-xL.

The ability of ATN-224 to induce cell death independent of Bcl-2/Bcl-xL status and degrade Mcl-1 suggests that ATN-224 has potential as an adjuvant to ABT-263 treatment. To determine whether ATN-224 enhances the effect of ABT-263, we combined low concentrations of ATN-224 with a low concentration of ABT-263. The combination of ATN-224 with ABT-263 resulted in an enhanced effect, in comparison to either drug alone, especially in those with high levels of Bcl-2 (Fig. 5C). These results suggest ATN-224 has potential as an adjuvant to drugs that target Bcl-2 and Bcl-xL.

## Discussion

Our data suggest that use of a copper chelator drug to target the mitochondria has potential as a therapeutic strategy to overcome apoptosis resistance and induce caspase-independent cell death in DLBCL. In cells with high levels of Bcl-2, Bcl-xL or Mcl-1, ATN-224 treatment causes mitochondrial dysfunction and induces the release of AIF from the mitochondria, resulting in nuclear condensation. ATN-224 treatment enhances the effect of ABT-263, which may be attributed to the ability of ATN-224 to degrade Mcl-1. These data suggest that ATN-224 has potential as an adjuvant with drugs that target Bcl-2 and Bcl-xL. The ability of ATN-224 to trigger caspase-independent cell death is an attractive alternative approach, either as a single agent or as an adjuvant, in the treatment of patients with defective caspase-dependent apoptotic pathways.

Targeting the non-canoncial function of Bcl-2 and other anti-apoptotic proteins is a successful strategy for circumventing apoptosis-resistance. Chen and Pervaiz showed that in response to cellular stress, cells with upregulated Bcl-2 maintain mitochondrial homeostasis, in comparison to cells without upregulated Bcl-2, by regulating the activity of CcOX (11). Other anti-apoptotic proteins, such as Bcl-xL and Mcl-1, have also been shown to maintain mitochondrial homeostasis by regulating mitochondrial respiration (8). The data suggest that the ability of these proteins to prevent cell death may be attributed to their ability to maintain mitochondrial homeostasis. CcOX, the terminal subunit of the mitochondrial respiratory chain, tightly controls *ΔΨ_m_* and is a target of ATN-224 (13,23,24). Here we show that ATN-224 treatment decreases *ΔΨ_m_*, independent of Bcl-2, Bcl-xL or Mcl-1 status, thus affecting their ability to maintain homeostasis. Recently, Ni Chonghaile et al showed that decreases in *ΔΨ_m_*, or mitochondrial ‘priming’, describes the proximity to death and correlates with better response and outcome (25). Our data suggest that the ability of ATN-224 to target the mitochondria and induce mitochondrial dysfunction is an attractive alternative approach to circumvent apoptosis-resistance and enhance the efficacy of cytotoxic agents, such as doxorubicin, vincristine or etoptoside (25).

Our data suggest that ATN-224 does not target the canonical function of the anti-apoptotic Bcl-2 family members. ATN-224 treatment results in loss of Mcl-1 either via inhibiting synthesis or promoting degradation via induction of Noxa, as seen in two of the cell types; Noxa specifically binds Mcl-1 resulting in degradation of the Mcl-1/Noxa complex (26). However, the release of cytochrome *c* or caspase dependence of the cell death mechanism have not been reported. Release of cytochrome *c* into the cytoplasm is a two step process (27). It requires release of cytochrome *c* from the outer face of the inner mitochondrial membrane into the intermembrane space and movement of cytochrome *c* into the cytoplasm through a pore formed in the outer membrane. Although the outer membrane may be compromised by ATN-224 treatment, our data on the reactive oxygen species-dependence of ATN-224-induced cell death suggest a mechanism by which cytochrome *c* would not be released into the intermembrane space. Release of cytochrome *c* from the inner membrane requires the peroxidatic activity of cytochrome *c* in the presence of H_2_O_2_ (28). ATN-224 inhibits SOD1, which converts superoxide to H_2_O_2_, resulting in increased superoxide and a decrease in H_2_O_2_ (data not shown). We have shown that the increased superoxide forms peroxynitrite and that ATN-224 induced cell death is peroxynitrite-dependent (12). Peroxynitrite forms nitrotyrosine residues on target proteins. In the presence of H_2_O_2_, cytochrome *c* is nitrated on Tyr74 which results in increased peroxidatic activity and triggers cytochrome *c* release and apoptosome formation (29). Under conditions of low H_2_O_2_, nitration occurs on alternate tyrosine residues resulting in cytochrome *c* that is unable to trigger downstream apoptotic events (29) and may quench the peroxidatic activity of cytochrome *c* (30).

Lack of cytochrome *c* release combined with the caspase-independence of cell death indicates that ATN-224 triggers cell death by a mechanism other than traditional apoptosis. The inability of cathepsin B, D and L inhibitors [indicators of lysosomal-induced cell death pathways (31)] or calpain inhibitors to attenuate cell death (data not shown) combined with release of AIF suggests that ATN-224 induces an alternative form of cell death. AIF is a mitochondrial-localized flavoprotein that translocates to the nucleus where it induces chromatin condensation and DNA degradation in a caspase-independent manner (32). AIF release can increase caspase activity; however, the AIF-induced cell death is caspase-independent (16). The expression of AIF is relatively high in DLBCL and is associated with a more favorable overall survival (OS) in patients treated with CHOP-like therapy (33). In this study, we found that ATN-224-induced cell death involves the release of AIF from the mitochondria. Our data suggest a mechanism by which ATN-224 could trigger AIF release. The mitochondrial permeability transition (MPT) pore is susceptible to oxidation by peroxynitrite, which could lead to the opening of the pore, allowing the release of AIF (34). The upregulation of anti-apoptotic proteins results in defective caspase-dependent apoptotic pathways. Many of the cytotoxic drugs used to treat DLBCL induce caspase-dependent apoptosis (3). The ability of ATN-224 to induce cell death via the release of AIF from the mitochondria suggests that patients with tumors that have defective caspase-dependent pathways could benefit from ATN-224 treatment.

Gene-deletion studies have demonstrated that the expression of Mcl-1 is critical to survival (35) and has been shown to correlate with high-grade follicular lymphomas, mantle cell lymphoma and DLBCL, predominately the ABC subtype (36–38). BH3 mimetics that target anti-apoptotic proteins are currently being developed and are in clinical trials (39). While those that target Bcl-2 and Bcl-xL have shown promise, resistance through the upregulation of Mcl-1 is a problem (7). BH3 mimetics that target Mcl-1 have been less successful due to their different binding affinities (6). Taken together, these data suggest that an alternative approach to target Mcl-1 is needed. In cells, Mcl-1 is tightly regulated and inactivated by different mechanisms that depend on the stimulus. For example, in response to oxidative stress, phosphorylation by JNK results in the loss of survival function; in dying cells, caspase-mediated cleavage results in the generation of a potent pro-apoptotic protein; and in response to genotoxic stress, poly-ubiquitylation results in proteasome-dependent degradation (40,41). Induction of Noxa is also a strategy to increase Mcl-1 degradation (26). While the mechanism by which ATN-224 treatment degrades Mcl-1 remains to be tested, our data suggest that ATN-224 could prove effective in tumors with increased Mcl-1.

In addition to *BCL2*, the upregulation of many other oncongenes, such as NF-κB, *MYC* and *BCL6*, occur in DLBCL and are associated with poor clinical outcome (42–45). The constitutive activation of NF-κB occurs in the more aggressive DLBCL ABC subtype (2). NF-κB is a redox sensitive transcription factor with known anti-apoptotic target genes, such as *BCL2* and *BCLXL* (46). A study by Pan *et al* has shown that ammonium tetrathiomolybdate treatment suppresses NF-κB transcription and decreases nuclear protein binding to the κB sequence (27). This suggests that NF-κB may be a target of ATN-224, however, this remains to be tested. The upregulation of *BCL2* can present with other complex karyotypes, which include upregulated *MYC* and/or *BCL6*, resulting in what is referred to as ‘double-hit’ and ‘triple-hit’ DLBCL (47). Double hit DLBCL with upregulated *BCL2* and *MYC* are characterized by highly aggressive clinical behavior and poor response to therapy (48). While double hit and triple hit DLBCL are rare, one retrospective study reported median survival of 6 and 4 months, respectively (49). Recently Quentmeier *et al* (21) reported that the U-2932 cells are actually two clones in one cell line. While both clones overexpress *BCL2*, one clone overexpresses *MYC* and the other *BCL6* (21). The sensitivity of the U-2932 cells to ATN-224 suggests that ATN-224 may also prove effective in tumors with increased *MYC* and/or *BCL6*.

In conclusion, our data indicate that ATN-224 has potential for the treatment of DLBCL. ATN-224 induces cell death in DLBCL cell lines that represent phenotypes that show characteristics of drug resistance and correlate with poor clinical outcome. The mechanism of action is different from many of the currently used therapies, which suggests that it could be used as a single agent or as an adjuvant in refractory disease. Tetrathiomolybdate has been used in clinical trials for the treatment of Wilson disease, a copper transport disorder (50), and for several tumor types other than lymphoma [(51–54) and references therein]. Our data combined with the safety data in the published clinical trials supports pursuing ATN-224 as a potential DLBCL chemotherapeutic.

## Figures and Tables

**Table I.** ATN-224 sensitivity.

NHL cell line	ATN-224 EC_50_ (nM)	ATN-224 EC_25_ (nM)
DLBCL		
SUDHL-4	105.61±7.39	53.56±4.14
SUDHL-8	9.73±0.84	4.94±0.64
SUDHL-10	32.71±2.04	15.16±1.33
U2932	29.06±0.22	15.24±0.50
MCL		
Granta 519	72.77±1.51	N/A

Values represent the mean ± SEM (n ≥3). N/A, not applicable.
